# Antioxidant, Anti-Proliferative Activity and Chemical Fingerprinting of *Centaurea calcitrapa* against Breast Cancer Cells and Molecular Docking of Caspase-3

**DOI:** 10.3390/antiox11081514

**Published:** 2022-08-03

**Authors:** Mourad A. M. Aboul-Soud, Hanane Ennaji, Ashok Kumar, Mohammad A. Alfhili, Ahmed Bari, Maqusood Ahamed, Mohamed Chebaibi, Mohammed Bourhia, Farid Khallouki, Khalid M. Alghamdi, John P. Giesy

**Affiliations:** 1 Chair of Medical and Molecular Genetics Research, Department of Clinical Laboratory Sciences, College of Applied Medical Sciences, King Saud University, P.O. Box 10219, Riyadh 11433, Saudi Arabia; malfeehily@ksu.edu.sa; 2 Laboratory of Chemistry-Biochemistry, Environment, Nutrition and Health, Faculty of Medicine and Pharmacy, Hassan II University of Casablanca, Casablanca B.P. 5696, Morocco; hanane.naj96@gmail.com; 3 Vitiligo Research Chair, Department of Dermatology, College of Medicine, King Saud University, Riyadh 11451, Saudi Arabia; aknirankari@gmail.com (A.K.); kmgderm@gmail.com (K.M.A.); 4 Department of Pharmaceutical Chemistry, College of Pharmacy, King Saud University, P.O. Box 2457, Riyadh 11451, Saudi Arabia; abari@ksu.edu.sa; 5 King Abdullah Institute for Nanotechnology, King Saud University, Riyadh 11451, Saudi Arabia; mahamed@ksu.edu.sa; 6 Biomedical and Translational Research Laboratory, Faculty of Medicine and Pharmacy, University of Sidi Mohamed Ben Abdellah, BP 1893, Km 22, Road of Sidi Harazem, Fez B.F. 1893, Morocco; mohamed.chebaibi@usmba.ac.ma; 7 Higher Institute of Nursing Professions and Technical Health, Laayoune 70000, Morocco; bourhiamohammed@gmail.com; 8 Biology Department, FTSE, Moulay Ismail University of Meknes, BP 609, Errachidia 52000, Morocco; f.khallouki@fste.umi.ac.ma; 9 Department of Dermatology, College of Medicine, King Saud University, P.O. Box 240997, Riyadh 11322, Saudi Arabia; 10 Toxicology Centre, University of Saskatchewan, Saskatoon, SK S7N 5B3, Canada; john.giesy@usask.ca

**Keywords:** breast cancer, purple star thistle, apoptosis, antioxidant potential, CG-MS profiling, caspase-3

## Abstract

*Centaurea calcitrapa* has been intensively utilized in ethnomedicinal practices as a natural therapeutic recipe to cure various ailments. The current study aimed to chemically characterize ethanolic extract of *C. calcitrapa* (EECC) aerial parts (leaves and shoots) by use of gas chromatography-mass spectrometry analyses (GC-MS) and investigate its antioxidant and in vitro anticancer activities, elucidating the underlying molecular mechanism by use of flow cytometry-based fluorescence-activated cell sorting (FACS) and conducting in silico assessment of binding inhibitory activities of EECC major compounds docked to caspase-3. CG-MS profiling of EECC identified a total of 26 major flavonoids and polyphenolic compounds. DPPH and ABTS assays revealed that EECC exhibits potent antioxidant activity comparable to standard reducing agents. Results of the proliferation assay revealed that EECC exhibit potent, dose-dependent cytotoxic activities against triple-positive (MCF-7) and triple-negative (MDA-MB-231) breast cancer cell models, with IC_50_ values of 1.3 × 10^2^ and 8.7 × 10^1^ µg/mL, respectively. The observed cytotoxic effect was specific to studied cancer cells since EECC exhibited minimal (~<10%) cytotoxicity against MCF-12, a normal breast cell line. FACS analysis employing annexin V-FITC/propidium iodide double labeling demonstrated that the observed anti-proliferative activity against MCF-7 and MDA-MB-231 was mediated via apoptotic as well as necrotic signaling transduction processes. The increase in fluorescence intensity associated with DCFH oxidation to DCF, as reported by FACS, indicated that apoptosis is caused by generation of ROS. The use of caspase-3-specific fluorogenic substrate revealed a dose-dependent elevation in caspase-3 substrate-cleavage activity, which further supports EECC-mediated apoptosis in MCF-7 cells. The major EECC compounds were examined for their inhibitory activity against caspase-3 receptor (1HD2) using molecular docking. Three compounds exhibited the highest glide score energy of −5.156, −4.691 and −4.551 kcal/mol, respectively. Phenol, 2,6-dimethoxy established strong binding in caspase-3 receptor of hydrogenic type, with residue ARG 207 and of PI-PI stacking type with residue HIS 121. By contract, hexadecenoic acid showed 3 H-bond with the following residues: ASN 615, ASN 616a and THR 646. Taken together, the current findings reveal that EECC exhibits significant and specific cytotoxicity against breast cancer cells mediated by the generation of ROS and culminating into necrosis and apoptosis. Further investigations of the phytoconstituents-rich *C. calcitrapa* are therefore warranted against breast as well as other human cancer cell models.

## 1. Introduction

Cancer is the second leading cause of mortality worldwide, after only cardiovascular illnesses, with a staggering reported incidence of 18.1 million new cases and 9.6 million deaths in 2018 [1]. Due to the inherent genetic heterogeneity leading to drug resistance, and despite significant advances in early diagnostic and novel treatment techniques, breast cancer (BC) remains one of the major causes of mortality among women worldwide [2]. It is globally ranked as the second diagnosed type of cancer, whereases it is the fifth cause in terms of cancer-associated fatalities [3]. While BC mortality rate is greater in low-Human Development Index (HDI) nations, its prevalence rate is more evident in economically developed nations [4]. This is most likely linked to such risk factors as smoking, alcohol, obesity and consumption of hormone-rich red meat [5]. BC is a malignant neoplasm that originates in epithelial tissue of the mammary gland. Annually BC affects more than 1.3 million women worldwide, of which the triple-negative (TNBC) or positive (TPBC) molecular subtypes garnered considerable research interest. The MDA-MB-231 cell line is classified as a human TNBC model that is characterized by absence of estrogen receptors (ERs), progesterone receptors (PRs) and human epidermal growth factor receptor 2 (HER2), which is collectively known as basal-like (ER-, PR-, HER2-). MCF-7 cells, are triple-positive (ER+, PR+, HER2+) breast cancer cells that are hormone-dependent and possess estrogen receptors on their cell surfaces [6]. Evidence from experimental and clinical studies has established critical involvement of estrogen in the proliferation and development of BC, by virtue of its binding to unique estrogen receptors alpha (ER-α) and beta (ER-β) [7].

One of the key mechanisms that is associated with the onset and continuation of BC progression is generation of reactive oxygen species (ROS) leading to enhanced expressions of oxidative stress markers and inhibition of the antioxidant enzymatic and non-enzymatic defense systems. ROS may have a role in early carcinogenesis by signaling such processes as fibroblast proliferation, epithelial hyperplasia and atypia leading to architectural deformation of the breast epithelial tissues. Base alteration events including formation of 8-hydroxydeoxyguanosine, which appears to be implicated in BC, have been detected at large concentrations in DNA isolated from BC patients [8].

BC is currently treated by use of diverse standard therapeutic strategies to control its spread and helpfully eliminate it completely from affected patients. These include surgery, hormone treatment and chemo-radio-therapy approaches whose severity, dose and duration are primarily dependent on size, histopathological and molecular tumor subtype, TNM staging, lymphadenopathy status and estrogen receptor status. To reduce the risk of metastasis and recurrence, synthetic chemotherapeutic agents are employed both pre- and/or post-surgically to combat cancer cells in BC patients. Despite effectiveness, synthetic chemotherapeutics suffer from a serious limitation that is low specificity, owing to their intrinsic adverse effects on non-target normal cells [1,9]. Hence, the urgent and growing demand for innovative, effective and low-cost anticancer treatments derived from natural, safer sources is justified. Medicinal plant-derived medicines have several advantages to synthetic chemical molecules, including less adverse effects, increased activity, lower cost and more accessibility. For a long time, plants have been employed as herbal drugs by humans. Well over 3000 medicinal herbs have been discovered as having antitumor properties, and thirty plant-derived natural products (NPs) have been evaluated in clinical trials against cancers [1]. The therapeutic effectiveness and low-toxicity of NPs as potent alternative therapeutic agents for cancer and other disorders have all led to their intensive and wide use [10].

The genus *Centaurea* is among the largest in the family Asteraceae, with 400–700 species. *Centaurea* species are distributed worldwide, but are mainly found in the Mediterranean and West Asian regions [11]. Specifically, there are 51 species and 24 varieties in Morocco alone. Moreover, it has been reported that Morocco is home to 16 taxa as well as 5 variants that are entirely native to the country [12]. *C. calcitrapa*, often called purple star thistle, is a biannual herbaceous perennial plant with a height of up to 60 cm. It is found throughout North Africa, Western Asia, northwestern India, and western and south-central Europe. It favors rocky areas, fertile ground and sunny and warm slopes, and grows along the roadside, in empty spots, as well as between rails [13].

For hundreds of years, several Centaurea species have been utilized in folk medicine as therapeutic ethnopharmacological agents against various ailments including *diabetes mellitus*, anorexia, diarrhea, inflammation, rheumatoid, fever, infection, menstrual disorders, vaginal candidiasis, skin diseases, opthalmia, jaundice, digestive conditions, microbial infections, inflammation and cancers [11,12]. Aerial parts of *C. triumfettii All.*, *C. urvillei* DC. spp. *stepposa* Wagenitz, *C. pullata* L. and *C. calcitrapa* L. are traditionally consumed as dietary supplements, usually either fresh or processed, as well as certain species being used to make drinks and tonics [11]. A recent survey performed in the eastern region of Morocco reported that ethnomedicinal use of *C. calcitrapa* is a common practice to treat diverse diseases and medical conditions [14].

The three-fold objectives of the current study were to: (i) investigate both the antioxidant and in vitro anticancer activities of chemically characterized ethanolic extract of *Centaurea calcitrapa* (EECC) aerial parts (leaves and roots), (ii) elucidate the EECC-mediated molecular mechanism underlying its cytotoxicity against breast cancer cells by use of FACS and (iii) conduct in silico analysis of its lead compounds for the assessment of their inhibitory activity against caspase-3, the executioner enzyme of apoptosis, by use of molecular docking.

## 2. Materials and Methods

### 2.1. Plant Material and Extraction Process

*C. calcitrapa* (Cc) were collected, during the flowering stage, in February 2020 from sandy habitat near Agadir City, southern Atlantic coast (29.602956 N −9.006312 W. Prof. Fennane Mohammed of the Scientific Institute in Rabat confirmed the authenticity of the plant, and the specimen was assigned the voucher code RAB112038. All parts of *C. calcitrapa* (leaves, shoots and roots) were washed separately with distilled water and dried under the electronic shade (FRITSCH 220V, 1500W) at room temperature (RT). After 96 h at RT, 50 g of plant materials were mixed with 350 mL of ethanol and extracted. The extract was concentrated using a rotary evaporator at 40–50 °C and stored at 4 °C until further use. To determine the percent yield, the following equation was employed:

$$\%\;\mathrm{yield}\;\mathrm{of}\;\mathrm{extract}=\frac{\mathrm{weight}\;\mathrm{of}\;\mathrm{extracted}\;\mathrm{material}\;}{\mathrm{weight}\;\mathrm{of}\;\mathrm{original}\;\mathrm{material}\;\mathrm{used}\;}\;\times \;100$$

### 2.2. Determination of Total Phenolic Content

The total phenolic content (TPC) in the ethanolic extract of *C. calcitrapa* was determined using the Folin–Ciocalteu reagent technique published by Dewanto et al. (2002) [15]. To 500 µL of extract, 1.5 mL of 10% Folin-Ciocalteu reagent was added and stirred for 5 min. Then 2 mL of 7.5% sodium carbonate solution (Na_2_CO_3_) was added and incubated at 37 °C for 2 h. Finally, using a UV-visible spectrophotometer, the absorbance was determined at 760 nm against a blank made up of the identical reagents as before except for the extract. The amount of total phenolic compounds was determined, using a gallic acid standard curve and represented as mg gallic acid equivalent (GAE) per g of dry weight of extract (mg GAE/g DW).

### 2.3. Determination of Total Flavonoids Content

The Total Flavonoids Content (TFC) was determined using the aluminum chloride (AlCl_3_) colorimetric technique published by Zhishen et al., (1999) [16]. In a nutshell, 200 µL of 2% AlCl_3_ solution was added to 200 µL of *C. calcitrapa* ethanolic extract and incubated for 30 min. The flavonoid concentration was determined using a quercetin standard curve and reported in milligrams of quercetin equivalents per gram of dry weight of extract (mg QuE/g DW).

### 2.4. Antioxidant Activity 

#### 2.4.1. DPPH● Radical Scavenging Assay

Radical scavenging activity of *Centaurea* against DPPH was measured spectrophotometrically, in a dark environment, using a previously reported technique) [17,18]. In ethanol, the samples were diluted at varying quantities (0.2–1.5 mg/mL). In 1950 µL of a DPPH ethanolic solution (2.3%), 50 µL of each diluted extract was added. After stirring the solution, it was put in the dark for 30 min at room temperature. The mixture’s absorbance was then measured at 517 nm and compared to the control (DPPH ethanolic solution). Ascorbic acid was used as a standard.

#### 2.4.2. Reducing Power (RP) Method

Antioxidant activity of *C. calcitrapa* extract was determined using RP assay [17,18] with slight modification. Ethanolic extract of *C. calcitrapa* was diluted using ethanol at various concentrations (0.2–1.5 mg/mL). The reference standard of RP assay using a gallic acid solution. Concentrations of reference standard solutions were 0.2–1.5 mg/mL. 1.25 mL phosphate buffer (0.2 M, pH 6.6) and 1.25 mL of 1% K_3_Fe CN)_6_ and reference standards were added to samples. The mixture was vortexed using a vortex shaker for 5 min then incubated for 30 min at 50 °C. After the incubation process, 1.25 mL of 10% TCA was added to the mixture and centrifuged (2000 rpm) for 10 min. As much as 0.625 mL of the supernatant—a result of the centrifugation process—was collected, and 0.625 mL of distilled water was added and then mixed well. To the mixture was added 0.125 mL of 1% ferric chloride until the colored solution was formed. The reference standard solutions and samples were determined using a UV-Visible Spectrophotometer at 700 nm. Gallic acid was used as the standard for comparison. The reaction mixture’s transmittance increased, indicating that the reducing power was growing.

#### 2.4.3. ABTS Assay

This test is based on the inhibition of the absorbance of the radical cation of ABTS that has a length characteristic absorption spectrum. After adding 1 mL of ABTS^+^ solution diluted to 10 µL of a sample, or gallic acid standard dissolved in ethanol at different concentrations (0.6–1 µg/mL), the reaction mixture was incubated for 6 min at 30 °C. Finally, the absorbance was determined exactly 6 min after the initial mixing for all samples at 734 nm. The absorbance of ABTS^+^ without the sample, the control, was measured daily. All measurements were repeated in triplicate. The percentage inhibition of ABTS^+^ sample was calculated according to the formula:

$$\mathrm{ABTS}\;\mathrm{radical}\;\mathrm{scavenging}\;\mathrm{activity}\;\%=\frac{\mathrm{Ac}-\mathrm{At}}{\mathrm{Ac}\;}\;\times \;100$$

where At and Ac are the respective absorbance of tested samples and ABTS^+^.

### 2.5. GC/MS Analysis of CCEE

Agilent GC 7890A combined with a triple-axis detector 5975 C having a single quadrupole mass spectrometer was used for GC-MS analysis. The chromatographic column used was an Agilent HP 5 MS column (30 m 0.25 mm 0.25 m film thickness) with a flow rate of 1 mL/min and high-purity helium as the gas carrier. Source temperature of MS was set at 230 °C and the Quad temperature was at 150 °C. Oven temperature was initially 40 °C (held for 1 min), then was increased to 150 °C at 5 °C min^−1^ (held for 1 min), then increased further to 300 °C at 5 °C min^−1^ for 1 min. MS ion source temperature was 150 °C and the inlet line temperature was 280 °C. The scan range was 40 to 600 amu at 70 eV (electron energy) and solvent delay of 3 min. Finally, unknown chemicals were identified by comparing spectra to the NIST 2008 database (National Institute of Standard and Technology library). Total time required for analyzing a single sample was 55 min.

### 2.6. Cell Lines and Culturing Conditions

Two human cancerous cell lines were used, mammary adenocarcinoma (MCF-7, ATCC^®^ HTB-22TM) and MDA-MB-231 breast cancer cells. Cell lines used in this work were obtained from the American Type Culture Collection (ATCC, Manassas, VA, USA) and stored at 150 °C in a freezing/storage solution containing 90% fetal bovine serum (FBS)/10% DMSO. To determine the selectivity of cytotoxic effect, the epithelial breast MCF-12 (ATCC^®^ CRL-10782™) cell line was included. DMEM/high glucose supplemented with two mM L-glutamine, 10% FBS, and 1% penicillin/streptomycin were used to cultivate cell lines. Afterward, sub-confluent cultures (80–90%) were trypsinized (Trypsin 0.05 percent/0.53 mM EDTA) and spilled according to the ATCC seeding ratio. All the cell lines were cultured at 37 °C in 5% CO_2_ in a humidified atmosphere. To acquire the appropriate dose, all of the samples were dissolved in DMSO and diluted in the inappropriate medium [1].

### 2.7. Cell Viability Assay

Cytotoxic effects of the ethanolic extract on viabilities of breast cancer cell lines under investigation were determined by assessing the ability to reduce enzymes present in viable cells to transform MTT into formazan crystals based on our previously published protocols [1,18]. The dose–response curves of ethanolic extract of *C. calcitrapa* for each cell line were established with escalating concentrations of 15.6, 31.25, 62.5, 125, 250, 500, and 1000 µg/mL. Concentrations causing 50% inhibition of growth of cells (IC_50_) were calculated by use of Excel trendline equation. The anticancer drug dasatinib, a potent multi-targeted kinase inhibitor of BCR-ABL and SRC family kinases, was employed as a positive control (data not shown).

### 2.8. Flow Cytometry

Control and treated cells were washed in PBS, suspended in Annexin-binding buffer, and stained with 1% Annexin-V-FITC and 20 ug/mL propidium iodide (PI) (Thermo Fisher Scientific, Waltham, MA, USA) for 20 min at RT in the dark. Following incubation, Annexin-V-FITC and PI fluorescence was detected by use of BD FACS Calibur (Betcon Dickinson, Franklin Lakes, NJ, USA) flow cytometer at 488/520 and 535/670 nm, respectively (PMID: 33387145) [1,19,20].

### 2.9. Reactive Oxygen Species (ROS) Assay

2′,7′-dichlorodihydrofuorescein diacetate (H_2_DCFDA; Thermo Fisher Scientific) was used to measure reactive oxygen species (ROS) as per (PMID: 33387145). Briefly, control and treated cells were washed in PBS and labeled with 10 uM H_2_DCFDA for 30 min at 37 °C away from light. Following repeated washing, DCF was finally excited by the blue laser at 488 nm and emitted fluorescence was captured at 520 nm [19].

### 2.10. Caspase Activity Assay

The fluorometric assay of the caspase-3 enzyme was examined using the 7-amido-4-trifluoromethylcoumarin (AFC) standard [21]. Activity of caspase-3 enzyme activity was represented as pmol AFC released/min/mg protein.

### 2.11. Molecular Docking of C. calcitrapa Compounds in Caspase-3 Active Site

The glide SP (standard precision) module of Schrodinger Maestro program was used to run ligprep, prepwizard, grid generation and docking calculations for the molecular docking investigation. Glide score, glide energy, glide emodel and glide ligand efficiency were identified as parameters.

#### 2.11.1. Ligand Preparation 

All compounds (ligands) were downloaded from PUBCHEM in SDF format. Then the ligands are prepared for docking calculations by the LigPrep tool in the Maestro 11.5 version of the Schrödinger Software program using the OPLS3 force field. A maximum of 32 stereoisomers were produced for the ligand after the ionization states at pH 7.0 ± 2.0 were selected.

#### 2.11.2. Protein Preparation 

The three-dimensional crystal structure of caspase-3 (PDB:3GJQ) was downloaded in PDB format from the protein data bank. The structure was then built and improved using Schrödinger-Maestro v11.5’s Protein Preparation Wizard. Hydrogens were added to the heavy atoms, selenomethionines were transformed to methionines, and all waters were removed. Minimization was done out using the force field OPLS3, with the maximum heavy atom RMSD (root-mean-square-deviation) set to 0.30 Å.

#### 2.11.3. Receptor Grid Generation

By clicking on any ligand atom, the creation module is launched, and a default grid box is created. The grid box had a volumetric spacing of 20 × 20 × 20, and the coordinates were x: 28.642, y: 34.169, and z: 12.047. Ligand was coupled to the grid box produced from protein using ‘Extra Precision’(XP). The XP GScore was used to evaluate the results.

#### 2.11.4. Glide Standard Precision (SP) Ligand Docking

SP flexible ligand docking was carried out in the glide of Schrödinger-Maestro v 11.5, within which penalties were applied to non-cis/trans amide bonds. For ligand atoms, the Van der Waals scaling factor and partial charge cutoff were set to 0.80 and 0.15, respectively. The final score was calculated using energy-minimized poses and displayed as a glide score. For each ligand, the best-docked pose with the lowest glide score value was recorded.

### 2.12. Statistics 

Graph Pad Prism 7 was used to conduct statistical analysis. The statistical significance of differences between two groups (EECC treatment and control) in biological experiments was assessed using a paired Student’s *t*-test.

## 3. Results

### 3.1. Total Flavonoids and Polyphenols Contents

The polyphenols content (TPC) of ethanolic extract of *C. calcitrapa* (EECC) was determined to be in the ranges of 35.52 to 44.72 mg GAE/g DW by use of the absorbance values of the solution according to its interaction with the Folin–Ciocalteu reagent, relative to standard solutions of gallic acid equivalents (y = 0.0064x − 0.1213, R^2^ = 0.9288) (Table 1). The total flavonoids content (TFC) was found to be in the range of 11.5 to 22.65 mg QuE/g DW, based on quercetin standard curve using the equation y = 0.0352x + 0.0273, R^2^ = 0.9889.

### 3.2. Antioxidant Capacity

To determine the concentration necessary to provide a 50% radical scavenging action, a series of concentrations were examined (IC_50_). According to the dosage-dependent linear standard curve of DPPH and ABTS radical-scavenging activity, EECC exhibited a lesser inhibitory potential than ascorbic acid, with an IC_50_ value of 0.84 ± 0.1 µg/mL and 0.86 ± 0.04 µg/mL EECC, compared to 0.037 ± 0.007 for ascorbic acid, respectively. Upon raising the concentration to 1.0 µg/mL, the scavenging activity of EECC then reached 52.6% and 65.5% in DPPH and ABTS, respectively, which was comparable to that of the standard. The EECC concentrations tested for reducing power (RP) were in the range of 0.6 to 1.0 µg/mL compared to ascorbic acid (Table 2). When compared to the standard, the RP values of EECC were significantly less in EECC. Calculated IC _50_ values were 0.88 ± 0.16 mg/mL for EECC compared to 0.012 ± 0.1 µg/mL for ascorbic acid.

### 3.3. Chemical Composition Profiling of Ethanolic Extract of Centaurea calcitrapa 

Phytochemical analysis by use of GC/MS has shown that EECC contains some lower and higher esters, terpenes, conjugated acid, fatty alcohol and various acids, as shown in the chromatogram (Supplementary Figure S1). GC/MS analysis of EECC revealed some interesting major constituents including aromadendrin and stigmasterol (10.3%), linoleic ester (10%) and ethyl oleate (10%). Some other bioactive phytoconstituents were identified in EECC including pyrolizidine alkalid, representing 2.7%. Moreover, other molecules including essential oil cadina (3.5%), conipheryl alcohol (5.1%), stigmasterol (10%), hexadecanoic acid (4.7%), ethylester of hexadecanoic acid (15%) and ethyl ester of octadecadienoic acid (2.5%) were also detected. Caryophyllene, another important bioactive sesquiterpene, was also detected in EECC, representing almost 1% of the total compounds present (Table 3).

### 3.4. Antiproliferative Effect of Ethanolic Extract of C. calcitrapa

**Figure 1:**
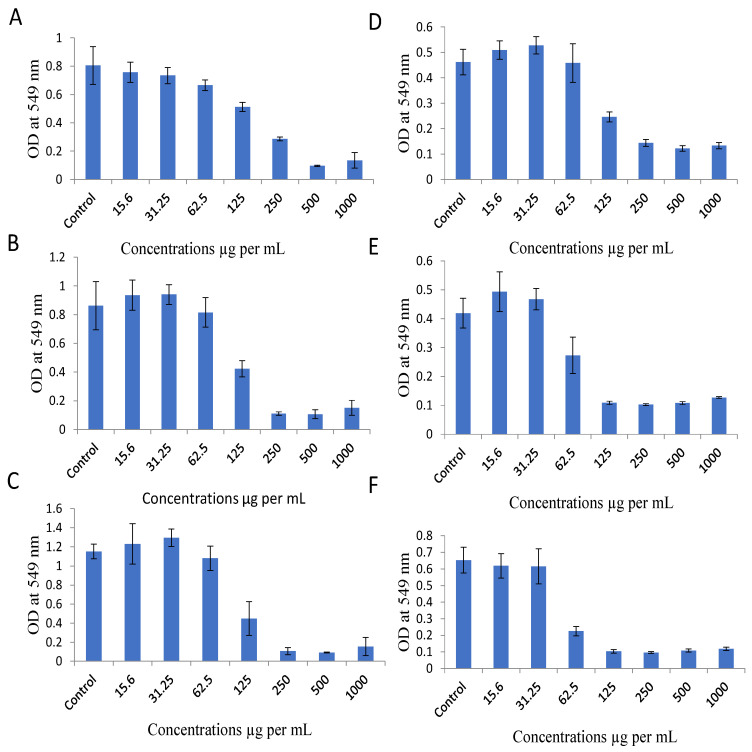
Dose- and time-dependent anti-proliferation activity of the ethanolic extract of *C. calcitrapa* (EECC). EECC inhibits proliferation of two breast cancer cells, MCF-7 ((**A**), for 24 h; (**B**), for 48 h; (**C**), for 72 h) and MDA 231 ((**D**), for 24 h; (**E**), for 48 h; (**F**), for 72 h). Cancer cells were treated with the indicated concentrations of EECC with DMSO control for 24, 48 and 72 h followed by determination of proliferation by MTT assay, as detailed in the Methods part. Data represent mean ± SD of 8 technical well-replicates.

To determine the cytotoxic activity of EECC on MDA-MB-231 and MCF-7 breast cancer cell lines, a standard MTT anti-proliferation assay was employed (Figure 1). Cells were tested with various concentrations (15.5, 31.25, 62.5, 125, 250, 500 and 1000 µg/mL) of EECC and the percentage of cells that survived was calculated compared to untreated cells (control). EECC was found to exhibit both dose- and time-dependent anti-proliferation activity. Concentrations resulting in 50% inhibition of growth of cancer cells (IC_50_) at 48 h post exposure to EECC, which were calculated by Excel trendline function [1], were 127.63 and 86.75 µg/mL for MCF-and MDA-MB-231, respectively. IC_50_ values indicate that the triple-negative MDA-MB-231 cells were more sensitive to EECC than the triple-positive MCF-7 one.

### 3.5. EECC Induces Apoptosis in Breast Cancer Cell Lines

**Figure 2:**
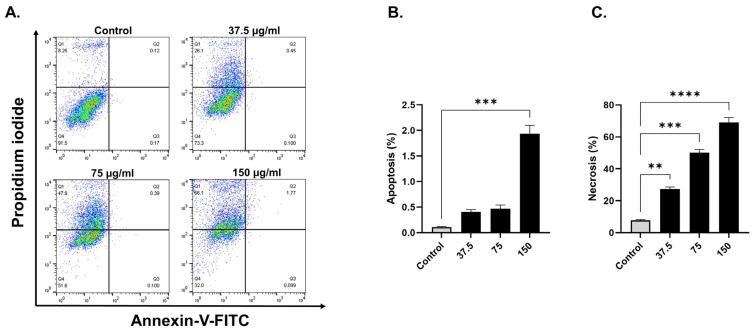
Evaluation of the apoptosis- and necrosis-inducing potential of ethanol extract of *C. calcitrapa* (EECC) in MCF-7. (**A**): FACS analysis of by Annexin V-FITC/PI double staining of MCF-7 cell after treatment with EECC at different concentrations for 48 h. EECC induced apoptotic and necrotic cell death in MCF-7cells. The Y-axis represents the PI-labeled population, whereas the X-axis represents the FITC-labeled annexin V positive cells. The four quadrants are representative of the following: Q1 quadrant (PI+/Annexin V−)—necrotic cells, Q2 quadrant (PI+/Annexin V+)—early apoptotic cells, Q3 quadrant (PI−/Annexin V+)—late apoptotic cells, Q4 quadrant (PI−/Annexin V−)—viable cells. (**B**,**C**): Cell population analysis (%) according to early apoptotic, late apoptotic and necrotic MCF-7 cells treated after exposure to EECC (37.5, 75 and 150 µg/mL), relative to non-treated control cells at different concentrations. ** *p* < 0.01, *** *p* < 0.001, **** = *p* < 0.0001 vs. control. Data represent mean ± SD of triplicates.

**Figure 3:**
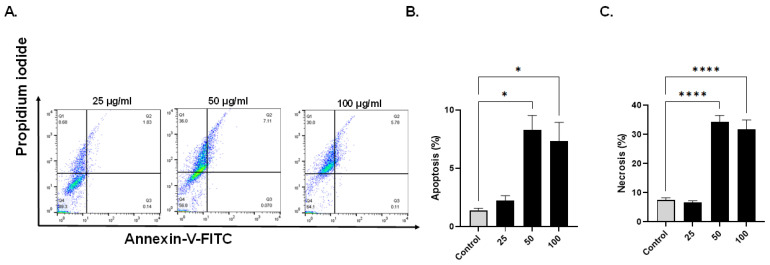
Evaluation of the apoptosis- and necrosis-inducing potential of ethanol extract of *C. calcitrapa* (EECC) in MDA-MB-231. (**A**): FACS analysis of by Annexin V-FITC/PI double staining of MDA-MB-231 cells after treatment with EECC at different concentrations for 48 h. EECC induced apoptotic and necrotic cell death in MDA-MB-231 cells. The Y-axis represents the PI-labeled population, whereas the X-axis represents the FITC-labeled annexin V positive cells. The four quadrants are representative of the following: Q1 quadrant (PI+/Annexin V−)—necrotic cells, Q2 quadrant (PI+/Annexin V+)—early apoptotic cells, Q3 quadrant (PI−/Annexin V+)—late apoptotic cells, Q4 quadrant (PI−/Annexin V−)—viable cells. (**B**,**C**): Cell population analysis (%) according to early apoptotic, late apoptotic and necrotic MDA-MB-231 cells treated after exposure to EECC (25, 50 and 100 µg/mL), relative to non-treated control cells at different concentrations. * *p* < 0.05, **** = *p* < 0.0001 vs. control. Data represent mean ± SD of triplicates.

Based on annexin V-FITC staining for detection of apoptosis, compared with control cells, breast cancer cells exposed to EECC for 48 h exhibited a significant increase in apoptosis (Figure 2 and Figure 3). A significant positive correlation was evident between dead cells stained with FITC-Annexin/PI for each EECC concentration and its corresponding IC_50_. MCF-7 breast cancer cells exposed to 37.5, 75 or 150 µg EECC/mL exhibited significant apoptosis as determined by FACS dual Annexin-V/PI staining, with the greatest effect observed for 150 µg EECC/mL (Figure 2A). Exposure to 150 µg EECC/mL resulted in significant (*p* < 0.001) apoptosis (0.11 + 0.01% to 1.93 ± 0.16%) (Figure 2A,B). At the same dose, there was also a significant (*p* < 0.0001) increase in the percentage of cells undergoing necrosis (Figure 2A,C) from 7.73 ± 0.52% in control cells to 69.10 ± 3.0%. In the case of MDA-MB-231, cells were also found to be susceptible to EECC exposure. While control values of 1.37 + 0.20% significantly (*p* < 0.05) increased to 8.32 + 1.21% for apoptotic cells following exposure to 50 µg/mL of EECC, a significant (*p* < 0.05) elevation to 7.35 + 1.59% with 100 µg/mL of EECC was observed (Figure 3A,B). The percentage of necrotic cells also increased significantly (*p* < 0.0001) from 7.44 + 0.78% in control cells to 34.33 + 2.18% (50 µg/mL) and to 31.67 + 3.28% (100 µg/mL), as observed in Figure 3A,C.

### 3.6. EECC-Induced Cell Death in Breast Cancer Cells Is Mediated through Oxidative Stress

**Figure 4:**
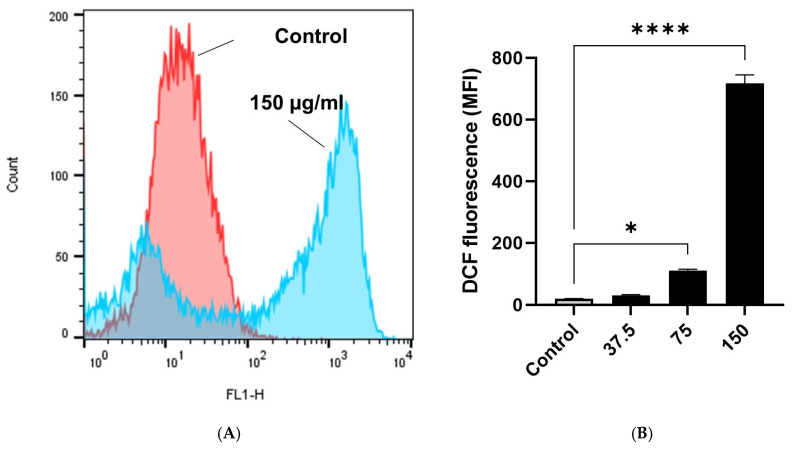
(**A**): Ethanol extract of *C. calcitrapa* (EECC) causes oxidative stress in MCF-7 cells. (**A**): Histogram represents relative DCF fluorescence intensity (FL1-H) vs. cell counts plot showing intracellular reactive oxygen species (ROS) generation in MCF-7 cells after 48 h exposure to EECC at the indicated concentration (150 µg/mL), relative to the control. (**B**): Quantitation of mean DCF fluorescence (ROS) in MCF-7 cell lines exposed to different concentrations of EECC (37.5, 75 and 150 µg/mL). * *p* < 0.05, **** = *p* < 0.0001 vs. control. Data represent mean ± SD of triplicates.

Overproduction of reactive oxygen species (ROS) predisposes cells to premature death. In order to assess whether intracellular levels of (ROS) instigate apoptosis and necrosis, MCF-7 cells were stained with 10 µM H_2_DCFDA, an oxidation-sensitive fluorescent probe, and subsequently exposed to EECC for 30 min. Significantly greater DCF fluorescence was observed in cells exposed to 75 (*p* < 0.05) or 150 (*p* < 0.001) µg EECC/mL, which ranged from 19.75 ± 0.45 a.u. to 110.50 ± 4.50 a.u. and to 717. 0 ± 28.0 a.u., respectively (Figure 4A,B).

### 3.7. EECC Induces Caspase-3 Enzymatic Activity

**Figure 5:**
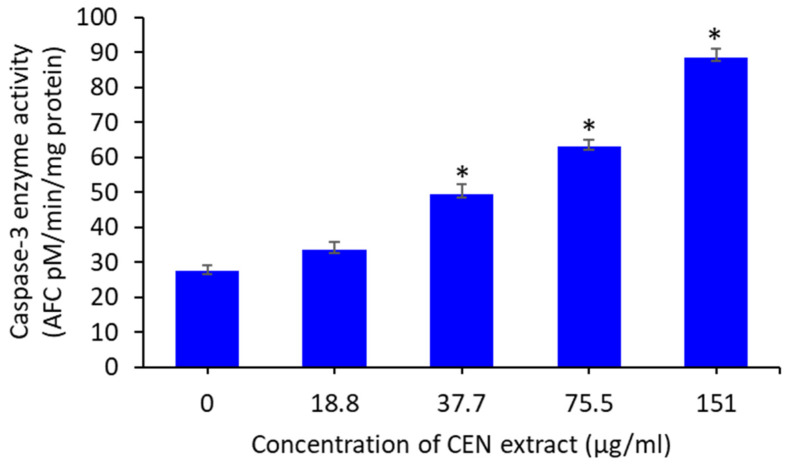
Caspase-3 enzyme activity of MCF-7 cells following exposure to different concentrations of ethanol extract of *C. calcitrapa* extract for 48 h. (*) denoted *p* < 0.05 relative to control. Data represent mean ± SD of 8 technical well-replicates.

Effects of ethanol extract of *C. calcitrapa* (EECC) on the activation of caspase-3 in MCF-7 cells were investigated, using the 7-amido-4-trifluoromethylcoumarin (AFC) standard fluorogenic probe. EECC resulted in significant (*p* < 0.05) activation of caspases-3 in MCF-7 cells at concentrations of 37.7, 75.5 and 151 µg/mL. However, caspase-3 enzymatic activity was small at the least dose of EECC (37.7 µg/mL), which was not significantly different from the control (Figure 5).

### 3.8. Molecular Docking of Caspase-3 with EECC Major Constituents

**Figure 6:**
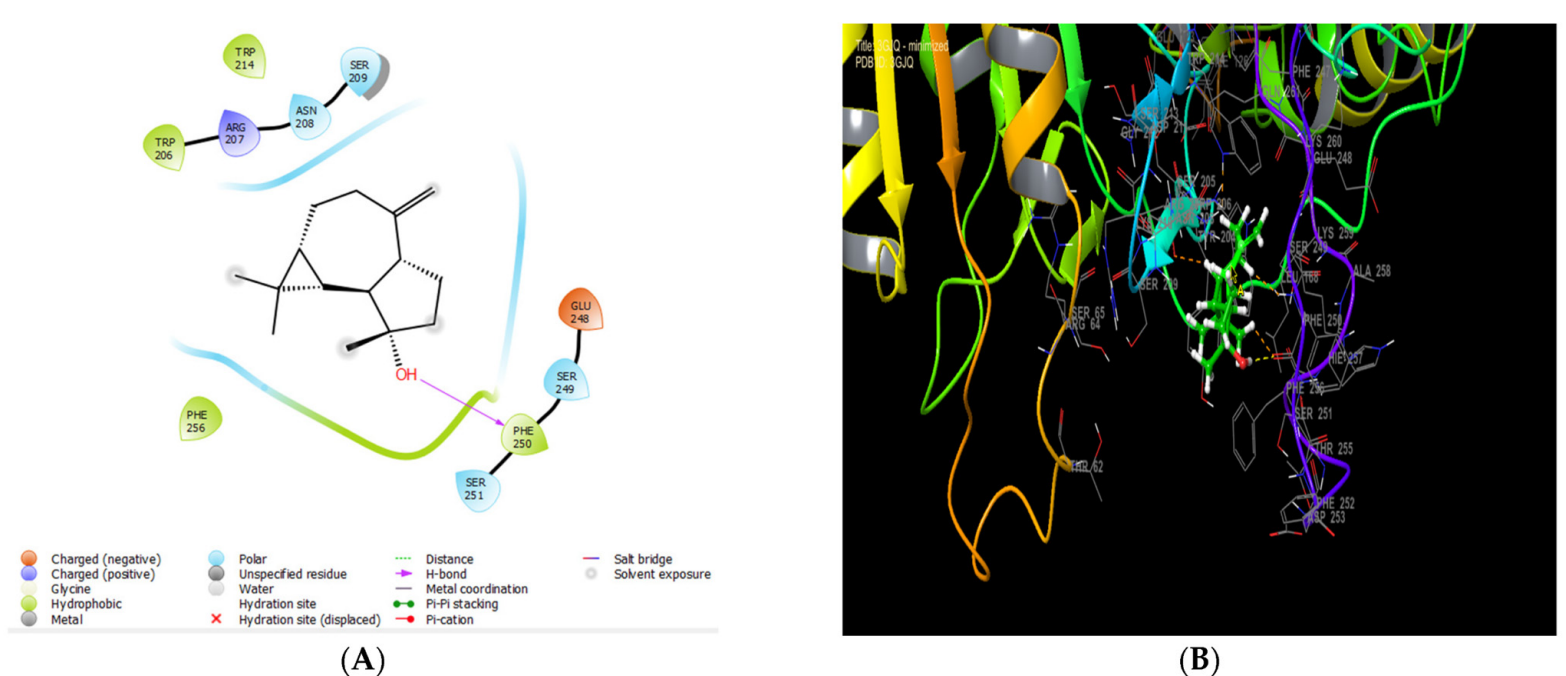
The 2D (**A**) and 3D (**B**) diagrams of the 1H-Cycloprop[e]azulen-7-ol, decahydro-1,1,7-trimethyl-4-methylene-, [1ar-(1a.alpha.,4a.alpha.,7.beta., 7a.beta.,7b.alpha.)]- interactions with the active site of caspase-3 (PDB: 3GJQ). The ligand-caspase glide score energy values were −5.156 kcal/mol.

To investigate their binding interaction of with different amino acids in the active site of caspase-3 (1HD2), seven of the major phytoconstituents found in EECC were docked. Cycloprop[e]azulen-7-ol, decahydro-1,1,7-trimethyl-4- methylene-, [1ar (1a.alpha.,4a.alpha.,7.beta., 7a.beta.,7b.alpha.)]-, 4-((1E)-3-Hydroxy-1-propenyl)-2-methoxyphenol and phenol, 2,6 dimethoxy- presented the largest glide score energy of −5.156, −4.691 and −4.551 kcal/mol, respectively. Furthermore, phenol, 2,6 dimethoxy- established strong binding in a caspase-3 receptor of hydrogenic type with residue ARG 207 and of PI-PI stacking type with residue HIS 121, while hexadecenoic acid showed 3 H-bond with the following residues: ASN 615, ASN 616 and THR 646 (Table 4) (Supplementary Figure S2). The greatest binding affinity in terms of glide score energy values was obtained with Cycloprop[e]azulen-7-ol, decahydro-1,1,7-trimethyl-4- methylene-, [1ar (1a.alpha.,4a.alpha.,7.beta., 7a.beta.,7b.alpha.)], with a value of −5.156 kcal/mol (Table 4 and Figure 6).

## 4. Discussion

Cancer is one of the leading causes of mortality worldwide. Chemotherapy and radiation, which are commonly used to treat cancer, have their own set of issues, such as limited selectivity for cancer cells or the establishment of drug resistance, which necessitates more study and treatment development [22]. Plant-derived natural products can be useful for discovery of novel, anticancer therapeutics [1,19,20,21,22,23]. To the author’s knowledge, this is the first report on antioxidant and antiproliferative activities of the Moroccan cultivar of *C. calcitrapa*. In this study, the ethanol extract of *C. calcitrapa* (EECC) exerted antioxidant activities. Moreover, EECC also exhibited dose- and time-dependent antiproliferative potencies against MCF-7 and MDA-MB-231 cells with IC_50_ values of 127.60 µg/mL and 86.75 µg/mL, respectively, 48 h post exposure. The antibacterial and in vitro cytotoxicity activities of Seribian *C. calcitrapa* leaves extracted by four solvents, methanol, 70% ethanol (EtOH), ethyl-acetate, 50% acetone and dichloromethane: methanol have been reported by Dimkić and co-workers [11]. They employed Orbitrap-mass spectrometry coupled to UPLC for the identification of a total of 55 phenolics and flavonoids (glycosides and aglycones) in the Serbian *C. calcitrapa* leaf extract, including centaureidin, jaceidin, kaempferide and nepetin [11]. The Serbian EECC extract exhibited potent antibacterial potencies, with minimum inhibitor concentration (MIC) values in the range of 1 µg/mL. Of note, EECC exhibited no cytotoxicity against MRC-5, a human fetal lung fibroblast cells, advocating its safe use a natural antibacterial agent. These finding are consistent with previous results reporting minimal cytotoxicity against MCF-12, a normal breast cell line which further supports its safe and specific use as a natural and potent anticancer agent. To the authors’ knowledge, this is the only report on the anticancer potency of ethanol aerial-parts extract of the Moroccan *C. calcitrapa*. In this context, Erol-Dayi and co-workers reported that methanol extract of *C. calcitrapa* exhibited dose-dependent antiproliferative potency against HeLa and Vero cells, cervical and kidney cancers, with IC_50_ values of 92.5 and 91.7 µg/mL, respectively [24].

Results of the FACS analysis revealed that the observed cytotoxicity of the Moroccan CCEE is mediated by apoptosis and necrosis that is signaled via ROS generation and caspa-3 activation. Binding to caspase-3 protein, reported in the current study, is indicative of the execution of the main intrinsic apoptotic signaling transaction pathway, which is communicated via the collapse of the mitochondrial membrane with Bax-triggered cytochrome c release and proteolytic activation of caspase-9 leading to engagement of caspase-3 [19,25]. Results reported here are consistent with those reported for extracts of *C. calcitrapa* collected in Italy, which revealed that it exhibits potent cytotoxicity (IC_50_ = 95 µg/mL) against U2OS, a human bone osteosarcoma epithelial cell, mediated by the induction apoptosis and caspase activation [22]. However, apoptosis induction is believed to be a mechanism during which several standard chemotherapeutic drugs function. Because cancer cells have acquired many survival strategies to escape apoptosis-inducing signaling molecules, the apoptosis pathway has been highlighted as a target for cancer prevention and treatment [26]. It has been reported that apoptosis is aggravated by the enhanced intracellular production of ROS. Naturally, cancer cells generate significantly more ROS levels than normal non-cancerous ones owing to their enhanced metabolic activity, which in turn leads to the activation of apoptosis in cancerous cells [27]. The EECC raised ROS levels in MCF-7 cells, indicating that ROS plays an essential role in MCF-7 cell death, according to our findings. Similarly, thymoquinone (TQ), the main active ingredient of *Nigella sativa*, has been reported to exert potent antiproliferative activities against both hepatocellular carcinoma and medulloblastoma cells in part by inhibiting NF-κB signaling by engaging both intrinsic and extrinsic apoptotic pathways in an ROS-dependent manner [19,28]. ROS are known to cause manifold and frequently inconsistent effects in cells [29]. If ROS molecules are not scavenged from tissues, they increase the risk of breast cancer development by causing nucleic acid, lipid and protein damage. ROS leads to an oxidative stress outbreak, which in turn causes cell cycle arrest and inflammation, culminating into apoptosis, necrosis, autophagy or consequences controlled by intersecting networks [30,31]. Moreover, it has been reported that *C. patula*, *C. pulchella* and *C. tchihatcheffii* extracts inhibit cell growth of A375 cells and cause apoptotic cell death in a caspase-dependent manner [32]. Other relevant reports revealed that the chloroform and ethyl acetate fractions of *C. bruguierana subsp. Belangerana* exhibit a potent cytotoxic effect against K562, AGS, MCF-7 and SW742 [33]. The extracts of *C. hermannii* have been reported to possess antiproliferative effects against HeLa cancer cells that are associated with induction of apoptosis and necrosis via the engagement of caspases 3, 7 and 9 [34]. Recently, it has been shown that the methanolic extract from *Centaurea albonitens* has a cytotoxicity activity against diverse hematologic malignant cell lines (human pre-B ALL cell lines (NALM-6 and REH), human APL cell line (NB4), human multiple myeloma cell line (KMM-1)), which are associated with the activation of apoptosis and upregulated caspase-3 activity [35].

*C. calcitrapa* is a species containing a wide range of phenolic compounds. Previous reports have confirmed the presence of flavonoids such as apigenin, luteolin, kaempferol, kaempferol 3-O-glucoside, eupatorin, jacaeosidin, neptin, protocathechuic acid and others [11]. Our GC/MS analysis showed that aromadendrine (10.3%), linoleic ester (10%) and ethyl oleate (10%) were present in large quantities, among other bioactive compounds, such as pyrrolizidine alkaloid (2.7%), as well as the sesquiterpene caryophyllene (1%), which might explain the antioxidant and anticancer potencies observed for the ethanol fraction. Because of their propensity to scavenge free radicals and active oxygen species such as singlet oxygen and hydroxyl radicals, polar solvents such as ethanol were often utilized to obtain the best extraction yields of phenolic compounds [36]. *C. calcitrapa* displayed a concentration-dependent scavenging activity (Table 2). The IC_50_ values found in the DPPH, ABTS, and RP assay revealed little differences with mean IC_50_ values of 0.84 ± 0.1 µg/mL, 0.86 ± 0.04 µg/mL and 0.879 ± 0.16 mg/mL, respectively. Other studies on *C. calcitrapa* species revealed IC_50_ values of 50 ± 3.8 μg/mL and 25.9 µg/mL using O_2_^•–^ scavenging activity [24,36]. Phenolic compounds belong to antioxidants, which act as free radical scavengers. The free radical scavenging activity frequently correlates with the total phenolic content in plants [37]. In this study, correlation between the phenolic compounds, antioxidant and anticancer potential of *C. calcitrapa* was confirmed.

Molecular docking (MD) is an efficient and trustworthy computational tool for predicting different binding affinities and modes as well as exploring the mechanisms of ligand binding between various molecules and target proteins. As a valuable tool to determine the mode of binding and strength of ligand–protein interactions, MD is frequently employed in the drug discovery and structural biology of natural products [38,39,40]. The interaction between proteins and ligands is also vital to take into account, and the interactions with the lowest binding energy are regarded as more stable. All three tested major constituents of EECC exhibited low (negative values) energy, which is indicative of the formation of ligand-caspase-3 complexes that are characterized by greater stabilities and higher binding affinities (highest glide score energy negative values in kcal/mol). Hence, results of the MD analysis confirmed that the observed EECC-mediated in vitro cytotoxicity against breast cancer cells proceeds via programmed cell death (PCD), also known as apoptosis.

## 5. Conclusions

Ethanolic extract of *C. calcitrapa* (EECC) exhibited potent and selective cytotoxicity against MDA-MB-231 MCF-7, triple-negative and triple-positive breast cancer cell line models, respectively. Based on our results, it is proposed that ROS-dependent engagement of necrosis and apoptosis as the main mechanism underlying the observed EECC-mediated cytotoxicity. The current study provides evidence that *C. calcitrapa*, which is consumed as a traditional ethnomedicinal product, possesses effective anticancer properties against breast cell lines (particularly MDA-MB-231 cells), which paves the road for future detailed investigation. However, having highlighted the promising in vitro anticancer potential of *C. calcitrapa*, more expanded studies on other in vitro cancer cell lines, pre-clinical studies on non-human primate models (NHPs) such as mouse and rats, and clinical trials are needed before considering it as an approved complementary and alternative medicine drug in clinical settings. Further in silico and virtual screening using molecular docking is also required to gain more insights into the effect EECC might have one other molecular targets in breast cancer cells.

## Figures and Tables

**Table 1:** Total phenolics and flavonoids content of ethanol extract of *C. calcitrapa.* Data represent mean ± SD of triplicates.

EECC Concentration (mg/mL)	TPC (mg GAE/g DW)	TFC (mg QuE/g DW)
**0.4**	35.52 ± 0.02	11.50 ± 0.04
**0.6**	37.10 ± 0.06	16.33 ± 0.03
**0.8**	44.72 ± 0.01	22.65 ± 0.03

**Table 2:** Scavenging activity of ethanol extract *C. calcitrapa*. Data represent mean ± SD of triplicates.

EECC Concentration (µg/mL)	DPPH %	ABTS %	RP (DO)
**0.6**	2.82 ± 0.08	17.7 ± 0.06	0.109 ± 0.006
**0.8**	32.82 ± 0.04	46.63 ± 0.02	0.225 ± 0.02
**1.0**	52.55 ± 0.05	65.52 ± 0.01	0.536 ± 0.06

**Table 3:** Chemical composition analysis of ethanol extract *C. calcitrapa*.

Compound Name	Mol Mass (amu)	RT (min)	Area (Ab × s)	% Area
*1H-Pyrazole, 3-ethyl-4,5-dihydro-1,4-dimethyl-*	126.116	17.942	565,985	0.91
*Phenol, 2,6-dimethoxy-*	154.063	20.01	715,956	1.15
*Caryophyllene*	204.188	21.645	618,120	1.1
*Phenol, 2-methoxy-4-(1-propenyl)-*	164.084	22.497	754,962	1.22
*Naphthalene, decahydro-4a-methyl-1-methylene-7-(1-methylethylidene)-, (4aR-trans)-*	204.188	23.025	443,165	0.71
*1H-Cycloprop[e]azulen-7-ol, decahydro-1,1,7-trimethyl-4-methylene-, [1ar-(1a.alpha.,4a.alpha.,7.beta.,7a.beta.,7b.alpha.)]-*	220.183	25.856	6,420,310	10.38
*Cyclohexane, 1,2-diethenyl-4-(1-methylethylidene)-, cis-*	176.157	25.996	1,091,401	1.76
*1,3,4-Thiadiazol-2-amine, 5-ethyl-*	129.036	28.954	462,954	0.75
*Ledene oxide-(II)*	220.183	29.381	687,616	1.11
*Cadina-1(10),6,8-triene*	202.172	29.667	2,217,882	3.58
*4-((1E)-3-Hydroxy-1-propenyl)-2-methoxyphenol*	180.079	30.049	3,116,064	5.01
*1-(2,4-Dimethylphenyl)-1-methylsiletane*	190.118	30.978	641,927	1.03
*Ethanone, 1-(2,4,6-trimethylphenyl)-*	162.104	31.798	1,537,594	2.5
*4-Allyl-5-pyridin-3-yl-2,4-dihydro-[1,2,4]triazole-3-thione*	218.063	32.403	1,107,187	1.8
*2,3,3-Trimethyl-2-(3-methylbuta-1,3-dienyl)-6-methylenecyclohexanone*	218.167	33.904	1,671,508	2.7
*n-Hexadecanoic acid*	256.24	34.75	2,925,123	4.74
*Hexadecanoic acid, ethyl ester*	284.272	35.056	9,264,578	15
*Linoleic acid ethyl ester*	308.272	38.198	6,864,898	11.1
*Ethyl oleate*	310.287	38.313	6,149,242	10.02
*Methyl 17-methyl-octadecanoate*	312.303	38.752	1,567,336	2.55
*Seneciphylline*	333.158	41.984	1,663,586	2.7
*Senecionine*	335.173	42.722	53,6060	0.86
*Octadecanoic acid, ethyl ester*	312.303	45.26	834,487	1.35
*7-Dehydrodiosgenin*	412,298	51.921	701,322	1.13
*dl-.alpha.-Tocopherol*	430.381	52.716	848,776	1.37
*Cholest-2-ene-2-methanol, (5.alpha.)-*	400.371	53.951	2,022,530	3.3
*Stigmasterol*	412.371	54.352	6,373,183	10.31

**Table 4:** Docking results with ligands in the caspase-3 (PDB:3GJQ) receptor.

Compound Name	Compound ID	Formula	Glide Score (kcal/mol)	Glide Energy (kcal/mol)	Glide Emodel (kcal/mol)
**Stigmasterol**	5280794	C_29_H_48_O	−4.458	−32.853	−40.504
**Phenol, 2,6 dimethoxy-**	7041	C_8_H_10_O_3_	−4.551	−20.673	−26.961
**Ethyl oleate**	5363269	C_20_H_38_O_2_	−2.947	−31.909	−35.326
**Hexadecanoic acid, ethyl ester**	12366	C_18_H_36_O_2_	0.827	−26.598	−29.745
**Linoleic acid ethyl ester**	5282184	C_20_H_36_O_2_	−3.482	−35.697	−40.818
**1H-Cycloprop[e]azulen-7-ol, decahydro-1,1,7-trimethyl-4-methylene-, [1ar-(1a.alpha.,4a.alpha.,7.beta., 7a.beta.,7b.alpha.)]-**	6432640	C_15_H_24_O	−5.156	−24.771	−19.457
**4-((1E)-3-Hydroxy-1-propenyl)-2-methoxyphenol**	1549095	C_10_H_12_O_3_	−4.691	−29.214	−22.927

## Data Availability

All data pertinent to this work are presented in the paper. Any requests should be directed to the corresponding author.

## Supplementary Materials

The supporting information can be downloaded at: www.mdpi.com/xxx/s1/, Figure S1: GC-MS chromatogram showing different phytoconstituents identified in ethanolic extract of *C. calcitrapa*. Image depicts a representative chromatograph of constituents identified by GC/MS in EECC. Peaks represent absolute abundances, whereas numbers on the x-axis represent retention times in min; Figure S2. The 2D and 3D diagrams of different polyphenolic compounds present in EECC and the active site of caspase-3.
