# Supplementary material for: Antioxidant, Anti-Proliferative Activity and Chemical Fingerprinting of Centaurea calcitrapa against Breast Cancer Cells and Molecular Docking of Caspase-3
Source: Antioxidants (Basel). 2022 Aug 3;11(8):1514. doi: 10.3390/antiox11081514 (PMC9405406; doi:10.3390/antiox11081514)
Supplement: Supplementary file 1 [file antioxidants-11-01514-s001.zip › antioxidants-1777157-supplementary.pdf]

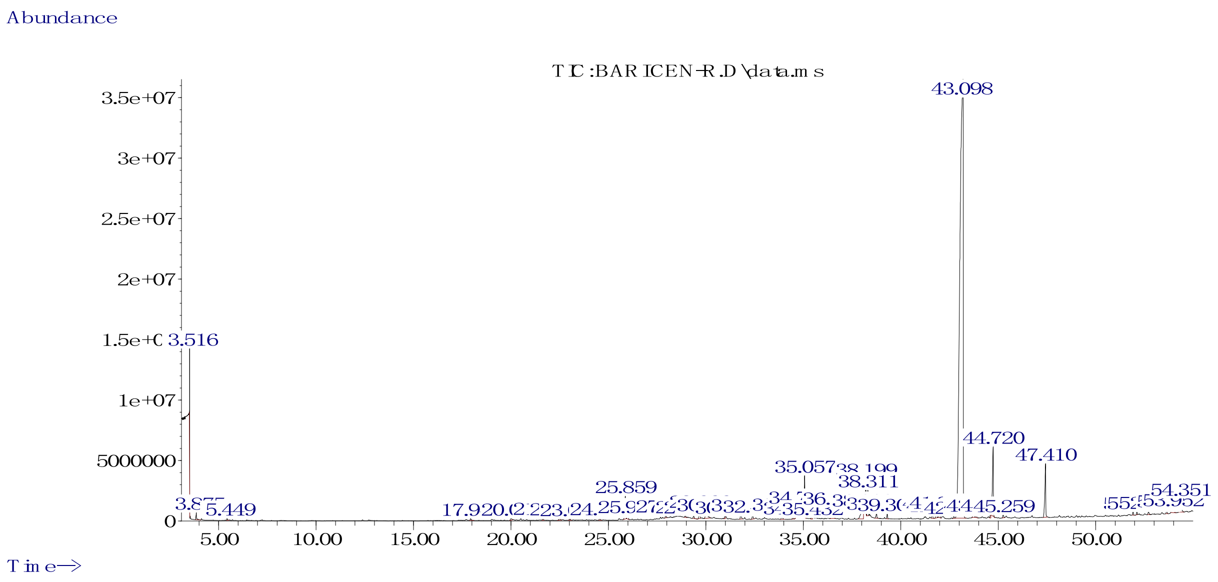

Supplementary Figure S1. GC-MS chromatogram showing different phytoconstituents identified in ethanolic extract of *C. calcitrapa*. Image depicts a representative chromatograph of constituents identified by GC/MS in EECC. Peaks represent absolute abundances, whereas numbers on the x-axis represent retention times in min.

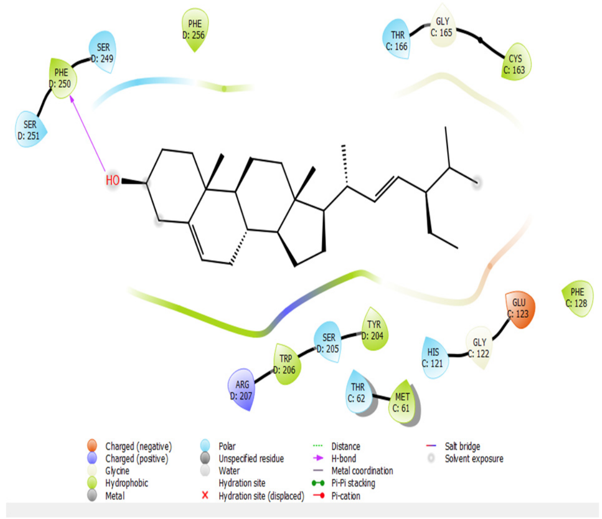

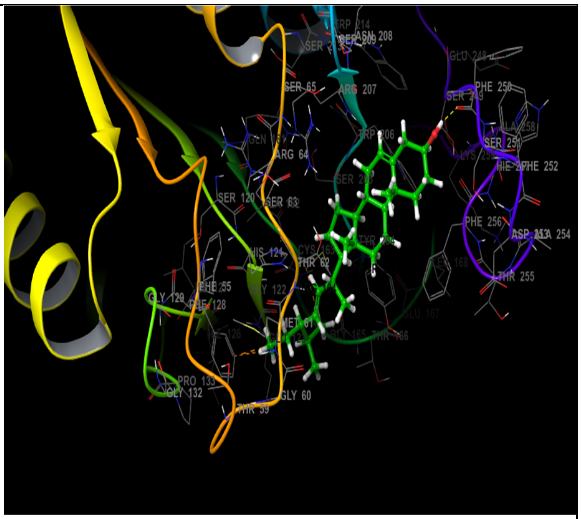

The 2D (a) and 3D (b) diagrams of the stigmasterol interactions with the active site of caspase-3 (PDB: 3GJQ)

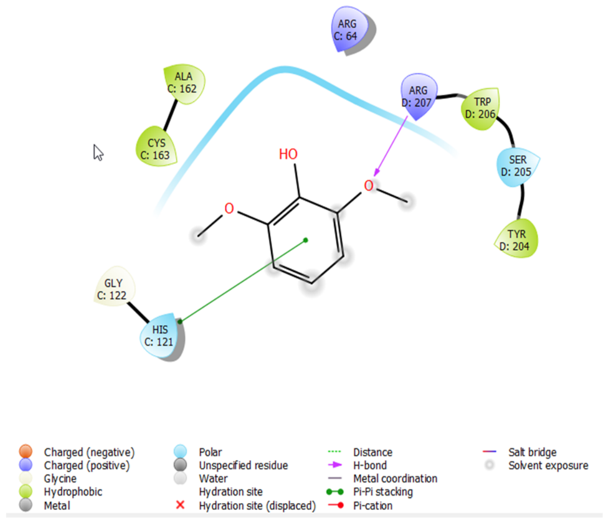

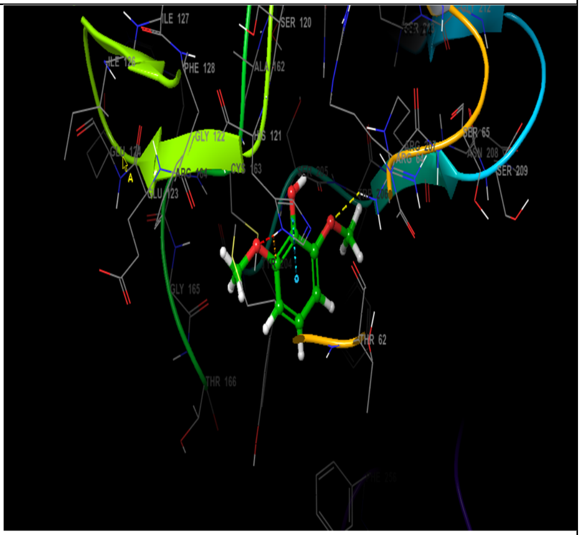

The 2D (a) and 3D (b) diagrams of the Phenol, 2,6 dimethoxy- interactions with the active site of caspase-3 (PDB: 3GJQ)

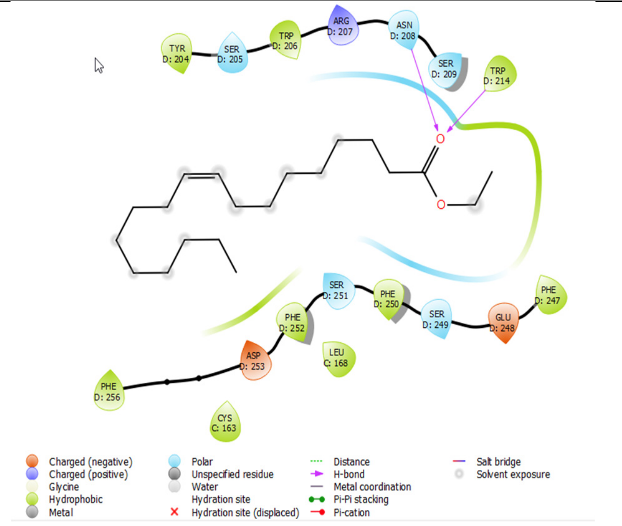

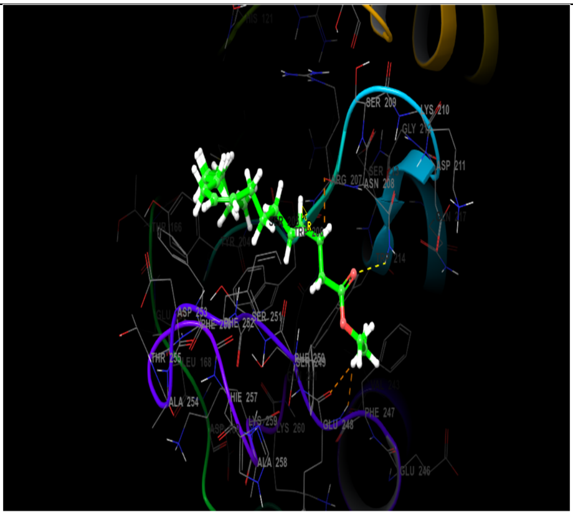

The 2D (a) and 3D (b) diagrams of the Ethyl oleate interactions with the active site of caspase-3 (PDB: 3GJQ)

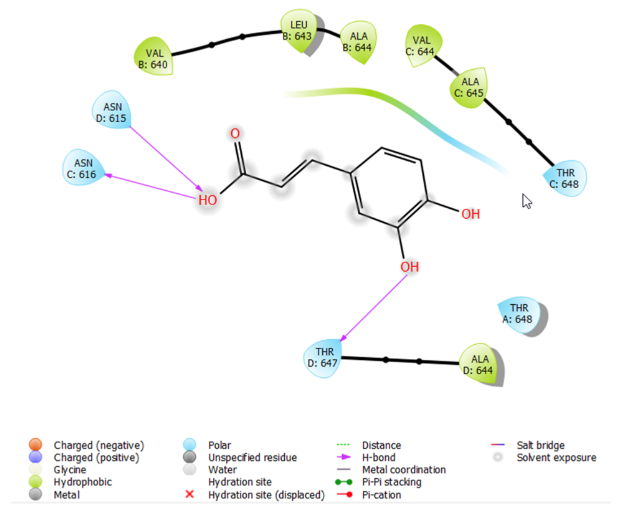

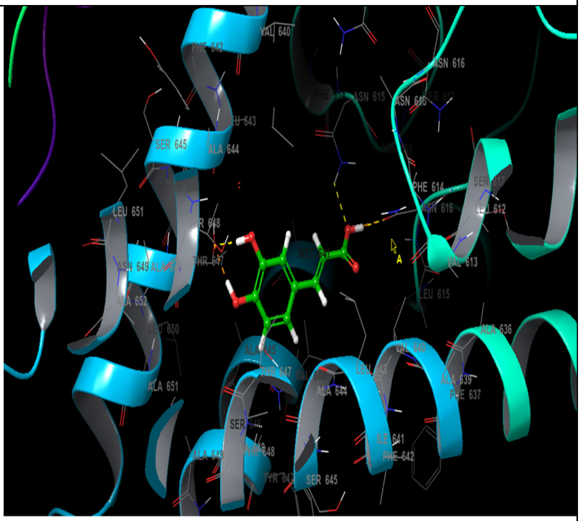

The 2D (a) and 3D (b) diagrams of the Hexadecanoic acid, ethyl ester interactions with the active site of caspase-3 (PDB: 3GJQ)

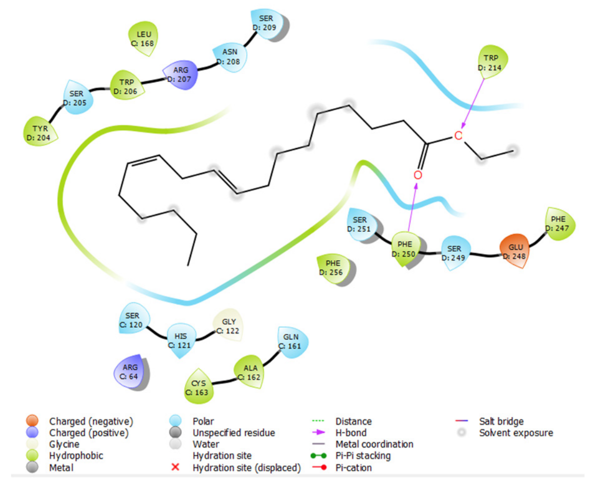

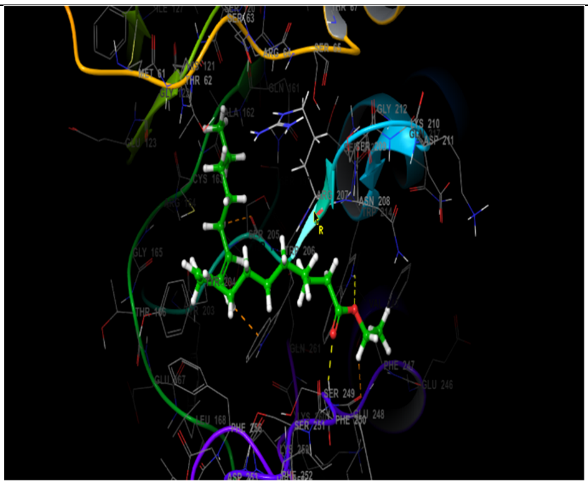

The 2D (a) and 3D (b) diagrams of the Linoleic Acid Ethyl Ester interactions with the active site of caspase-3 (PDB: 3GJQ)

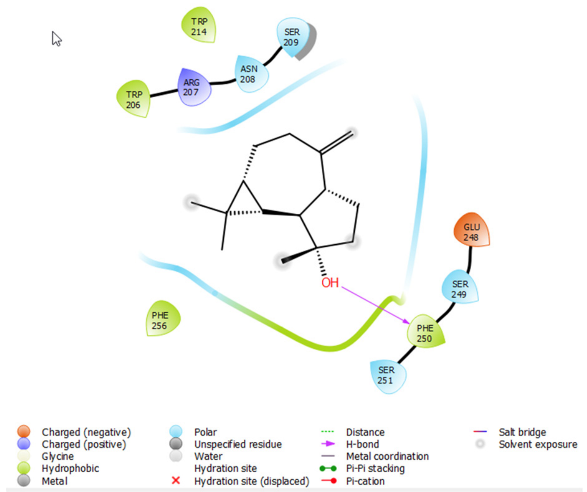

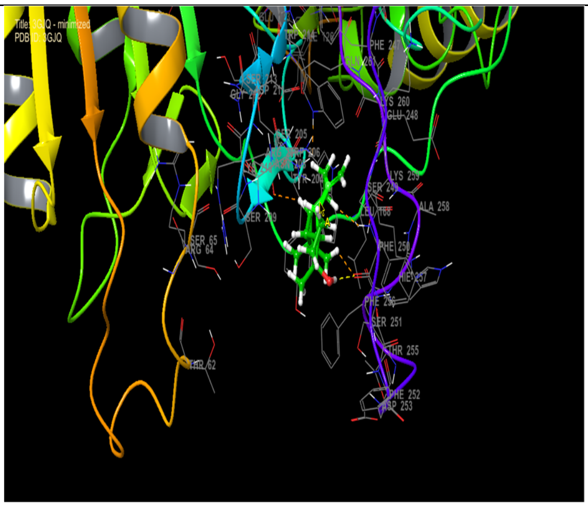

The 2D (a) and 3D (b) diagrams of the 1H-Cycloprop[e]azulen-7-ol, decahydro-1,1,7-trimethyl-4-methylene-, [1ar-(1a.alpha.,4a.alpha.,7.beta., 7a.beta.,7b.alpha.)]- interactions with the active site of caspase-3 (PDB: 3GJQ)

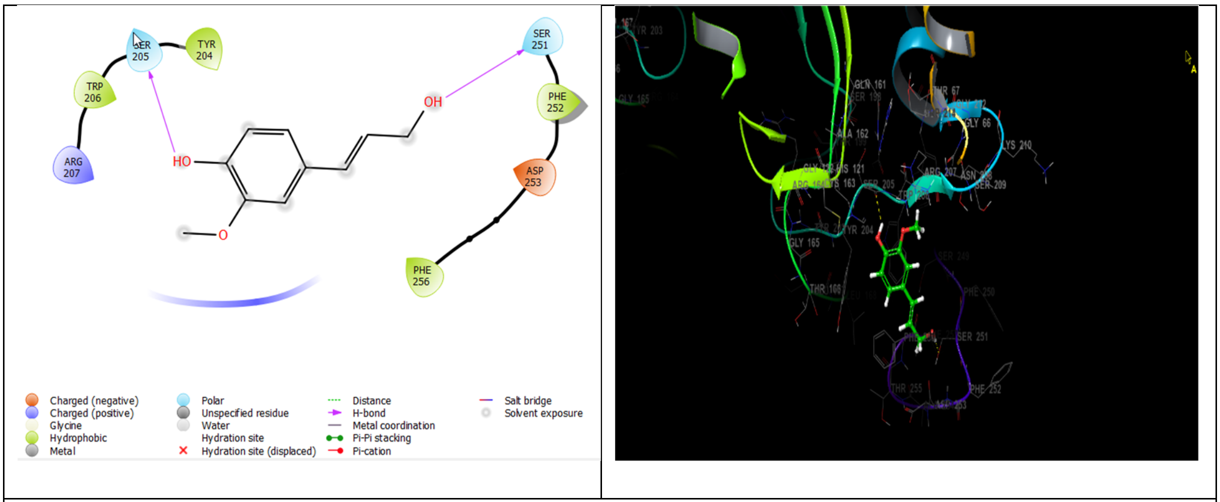

The 2D (a) and 3D (b) diagrams of the 4-((1E)-3-Hydroxy-1-propenyl)-2-methoxyphenol interactions with the active site of caspase-3 (PDB: 3GJQ)

Supplementary Figure S2. The 2D and 3D diagrams of different polyphenolic compounds present in EECC and the active site of caspase-3.
